# Histamine-Producing Intestinal Dysbiosis and Its Role in Lower Urinary Tract Infections and Irritable Bowel Syndrome in Young Women

**DOI:** 10.3390/nu18010016

**Published:** 2025-12-19

**Authors:** Florina Ruța, Călin Avram, Elena Mardale, Raluca Maior, Cristina Filip, Sebastian Nemeth

**Affiliations:** 1 Department of Community Nutrition and Food Safety, George Emil Palade University of Medicine, Pharmacy, Science and Technology of Targu Mures, 540139 Targu Mures, Romania; florina.ruta@umfst.ro (F.R.); raluca.maior@umfst.ro (R.M.); 2 Department of Medical Informatics and Biostatistics, George Emil Palade University of Medicine, Pharmacy, Science and Technology of Targu Mures, 540139 Targu Mures, Romania; 3 Doctoral School of Biomedical Sciences, University of Oradea, 410087 Oradea, Romania; 4 Biochemistry and Environmental Chemistry, George Emil Palade University of Medicine, Pharmacy, Science and Technology of Targu Mures, 540139 Targu Mures, Romania; cristina.filip@umfst.ro; 5 Department of Pharmacy, Faculty of Medicine and Pharmacy, University of Oradea, 410087 Oradea, Romania; snemeth@uoradea.ro

**Keywords:** dysbiosis, irritable bowel syndrome, urinary tract infections, histamine-producing gut flora, diet

## Abstract

**Background:** Lower urinary tract infections (LUTIs) and irritable bowel syndrome (IBS) have been associated with histamine-producing gut dysbiosis, characterized by an overgrowth of histaminogenic bacteria and opportunistic fungi. This study examines the relationship between histaminogenic microbial imbalance, dietary factors, and LUTIs in women with IBS, emphasizing the potential nutritional contributions to microbiota modulation. **Methods:** A cohort of 188 women was evaluated by analyzing the intestinal microbiota associated with sporadic and recurrent lower urinary tract infections and irritable bowel syndrome, along with a questionnaire on risky eating behaviors. **Results:** Dysbiosis was associated with an overgrowth of histamine-producing bacteria (*Escherichia coli*, *Enterobacter* spp., *Clostridium* spp.) and *Candida albicans*, along with a depletion of protective taxa (*Lactobacillus*, *Bifidobacterium*). Dysbiosis, characterized by an increase in histamine-producing bacteria (*Escherichia coli*, *Enterobacter* spp., *Clostridium* spp.) and *Candida albicans*, together with a depletion of protective taxa (*Lactobacillus*, *Bifidobacterium*), has been associated with lower urinary tract infections and irritable bowel syndrome. Regarding the increase in histaminogenic flora, significant associations with dietary factors concerned only the frequent consumption of fast food. **Conclusions:** These findings highlight the role of histamine-driven dysbiosis in sustaining chronic inflammation and susceptibility to LUTIs and IBS, supporting microbiota modulation as a potential preventive and therapeutic strategy.

## 1. Introduction

Gut microbiota and systemic health. The intestinal microbiota plays a crucial role in maintaining digestive, metabolic, and immune homeostasis. Its imbalance, known as dysbiosis, has been increasingly associated with a wide spectrum of systemic disorders. Du et al. emphasized the influence of dietary habits on gut microbial composition and the indirect link with urinary tract infection (UTI) risk, suggesting that diet may modulate susceptibility to such infections through ecosystem-level microbial shifts [1]. Similarly, Dominoni et al. highlighted the interplay between the urogenital microbiota and the urothelial immune microenvironment, linking microbial alterations to recurrent cystitis [2]. Brigida et al. further proposed that modulation of the intestinal microbiome could serve as a therapeutic strategy for the prevention and management of recurrent UTIs [3]. In the context of irritable bowel syndrome (IBS), Shrestha et al. summarized evidence confirming the contribution of microbial imbalances to disease pathogenesis and symptom expression, supporting the hypothesis of a shared dysbiotic background between IBS and UTIs [4]. Recent urinary microbiome studies have also shown that alterations in bacterial communities may promote persistence and recurrence [5].

Histamine-producing bacteria. Histamine also plays a key role in regulating mucosal permeability and inflammation through the link between increased permeability and systemic inflammation driven by dysbiosis [6,7]. The expansion of histamine-producing bacterial populations, accompanied by the depletion of protective species, such as *Lactobacillus* and *Bifidobacterium*, can create a pro-inflammatory intestinal environment, and dietary interventions can significantly reduce the abundance of histamine-secreting bacteria [8].

Fungal dysbiosis. Overgrowth of *Candida albicans* has been described in the context of intestinal dysbiosis and is associated with inflammatory disorders and carcinogenesis [9,10]. Opportunistic fungi, together with histamine-producing bacteria, contribute to the maintenance of a dysbiotic intestinal environment that favors both symptoms related to irritable bowel syndrome and increased susceptibility to recurrent urinary tract infections.

Diet. Microbial metabolites derived from the diet can modulate intestinal metabolism and the synthesis of bioactive compounds, including histamine [11,12], with influence also on the serum metabolite profile, negatively impacting clinical parameters [13]. Nutritional factors can indirectly modulate the intestinal balance by altering the substrates available for bacterial fermentation and metabolism, thus affecting the production of bioactive metabolites such as histamine [11,12,13].

Consequently, exploring the interrelationship between diet, histaminogenic flora, and dysbiosis may provide novel insights for the prevention and integrated management of IBS and recurrent UTIs.

The aim of this study was to investigate the associations between intestinal microbiota composition (bacterial, fungal, and histaminogenic communities), dietary habits, and fluid intake in relation to the presence and recurrence of lower urinary tract infections (LUTIs) in women with irritable bowel syndrome (IBS), with particular focus on the role of opportunistic and histaminogenic microorganisms, fungi, and protective taxa such as *Lactobacillus* and *Bifidobacterium*.

## 2. Materials and Methods

Selection of the Study Group. Women aged 18–45 years were recruited from family medicine practices and private dietetics clinics in the Mureș region, Romania. Eligibility was based on a medical history of lower urinary tract infections (LUTIs) and willingness to provide stool and urine samples.

To investigate the associations between gut microbiota composition, lower urinary tract infections, and irritable bowel syndrome (IBS), participants were stratified into clinical subgroups as follows: women without a history of LUTIs or IBS (NLUTIs, NIBS); women with sporadic LUTIs (SLUTIs), defined as one or two symptomatic LUTI episodes within the previous 12 months; women with SLUTIs and NIBS; women with SLUTIs and IBS; and women with recurrent LUTIs (RLUTIs), characterized by at least three and up to more than six symptomatic LUTI episodes within the past 12 months. This stratification allowed the assessment of how lower urinary tract infections, with or without the presence of irritable bowel syndrome, are associated with intestinal dysbiosis.

Inclusion criteria were as follows: women aged 18–45 years, a documented history of lower urinary tract infection (LUTIs) confirmed by urine culture (for the LUTIs subgroups), availability of dietary and hydration data, and provision of written informed consent for biological sample collection. Only women were included in order to reduce variability related to sex-specific differences in the urogenital microbiota and to reflect the epidemiology of LUTIs, which predominantly affect otherwise healthy young women.

Exclusion criteria included recent use of antibiotics, probiotics, or antifungal agents within the previous 30 days; use of other medications potentially affecting microbiota (e.g., contraceptives, proton pump inhibitors); pregnancy or lactation; chronic gastrointestinal diseases; metabolic or endocrine disorders; renal or hepatic insufficiency; ongoing immunosuppressive therapy; or inability to provide informed consent. Medication use was assessed by self-report from participants.

Questionnaires and Dietary Assessment

Dietary habits history was assessed using a structured questionnaire with a 5-point Likert scale (1 = strongly disagree, 5 = strongly agree). Participants reported consumption frequency for predefined food categories, with portion sizes standardized. Data allowed the association of dietary patterns with microbiota composition. Dietary intake was assessed using the shortened, validated version of the Rapid Eating Assessment for Participants (REAP-S) [14].

Urinary Tract Infection Assessment

Data regarding lower urinary tract infections (LUTIs) were obtained from participants’ primary care medical records, based on laboratory urine analyses performed by certified clinical laboratories. LUTIs were considered confirmed if the medical records documented the presence of ≥10^3^ colony-forming units (CFU)/mL of uropathogenic bacteria. These records were used to classify participants into groups with SLUTIs (<3 symptomatic episodes in the past 12 months) and recurrent RLUTIs (≥3 symptomatic episodes in the past 12 months).

Irritable Bowel Syndrome Assessment

Women with a registered IBS diagnosis in primary care records, confirmed by a gastroenterology specialist using the Rome IV criteria, were included in the study.

Microbiota Analysis

Fecal samples (1 g) were collected at least 7 days after the completion of any antibiotic therapy, stored at 2–8 °C, and processed within 5 days of collection. Culture-based analysis quantified pathogenic bacteria (*Escherichia coli*, *Klebsiella* spp., *Enterobacter* spp., *Clostridium* spp.), fungi (*Candida* spp.), and beneficial bacteria (*Lactobacillus* spp., *Bifidobacterium* spp.). Dysbiosis was assessed as mild (1–5), moderate (6–12), or severe (>12), with values ≥6 indicating clinically relevant dysbiosis. Microbiota analysis relied on culture-based methods, capturing only the cultivable fraction of the community (CCM). All procedures were performed by trained personnel in accordance with laboratory biosafety standards, and participant anonymity was strictly maintained.

The distribution of histaminogenic flora was further analyzed across clinical subgroups, including the entire study cohort, women with no lower urinary tract infections (LUTIs), sporadic LUTIs, recurrent LUTIs, and those with or without irritable bowel syndrome (IBS). Comparative analyses were also performed according to microbial and biochemical parameters, putrefactive flora, fecal pH, acidifying flora, and fungal counts to elucidate the interactions between gut microbial ecology and histamine metabolism. Normal parameters of histaminogenic flora were classified as NHF (normal histaminogenic flora), whereas elevated levels were classified as IHF (increased histaminogenic flora).

### Statistical Analysis

The study database was created in Microsoft Excel 2021, and statistical analyses were performed using GraphPad Prism version 10. For continuous numerical variables, means and standard deviations were calculated, while categorical variables were summarized as counts and percentages. Associations between the presence of urinary tract infections (LUTIs) or irritable bowel syndrome (IBS) and the analyzed bacterial taxa were assessed using the chi-square test. In addition, logistic regression analyses were performed to identify associations between participants without LUTIs versus those with SLUTIs, between those with SLUTIs versus those with RLUTIs, and between those diagnosed with irritable bowel syndrome associated with SLUTIs and those without IBS.

A 95% confidence interval was applied, and *p*-values < 0.05 were considered statistically significant.

Ethical Considerations. The study was conducted between November 2022 and November 2023. All participants, recruited on a voluntary basis through family medicine and dietetics clinics, provided written informed consent. The protocol was approved by the local Ethics Committee and adhered to the Declaration of Helsinki.

## 3. Results

Of the 188 participating patients, 62.2% (*n* = 117) had sporadic lower urinary tract infections (SLUTIs), defined as 1–2 episodes in the past year; 27% (*n* = 52) had recurrent lower urinary tract infections (RLUTIs), defined as 3 or more episodes in the past year; and 10% (*n* = 19) reported no LUTIs during the past year. Among patients with SLUTIs, 40.1% (*n* = 47) also had irritable bowel syndrome (IBS).

Using culture-based methods, sporadic urinary tract infections were significantly associated with *Escherichia coli* (*p* = 0.0143), *Bifidobacterium* spp. (*p* < 0.0001), *Lactobacillus* spp. (*p* = 0.0203), *Candida albicans* (*p* < 0.0001), and *Candida* spp. (*p* < 0.0001), whereas no significant associations were observed for *Klebsiella* spp., *Proteus* spp., *Enterobacter* spp., *Clostridium* spp., or *Geotrichum* spp. (*p* > 0.05). Recurrent urinary tract infections were significantly associated only with *Bifidobacterium* spp. (*p* < 0.0001) (Table 1).

Logistic regression comparing SLUTI and NLUTI patients revealed significant associations with increased *Escherichia coli* (*p* = 0.0357) and *Candida albicans* (OR = 3.48, *p* = 0.0123) (Table 2).

Logistic regression showed that recurrent lower urinary tract infections (RLUTIs) were significantly associated only with reduced *Lactobacillus* spp. (OR = 1.78, *p* = 0.0228), while other microbial changes, including *Escherichia coli*, *Klebsiella* spp., *Clostridium* spp., *Bifidobacterium* spp., and *Candida albicans*, were not significant (Table 3).

In patients with SLUTIs and IBS, culture-based analysis (CCM) revealed significant differences for *Escherichia coli* (*p* < 0.0001), *Enterobacter* spp., *Clostridium* spp. (*p* = 0.006), *Bifidobacterium* spp. (*p* < 0.0001), *Lactobacillus* spp. (*p* = 0.0224), *Candida albicans* (*p* < 0.0001), and *Candida* spp. (*p* < 0.0001) (Table 4).

Statistical analysis revealed strong associations between histaminogenic flora and specific gut microbiota profiles. Significant differences were observed in the CCM for *Escherichia coli* (*p* < 0.0001), *Klebsiella* spp. (*p* < 0.0001), and *Enterobacter* spp. (*p* < 0.0001) (Table 5).

Statistical analysis revealed significant associations between elevated histaminogenic flora and intestinal fungal colonization. Significant differences were observed in the CCM for *Candida albicans* (*p* < 0.0001), *Candida* spp. (*p* < 0.0001), *Geotrichum* spp. (*p* < 0.0001), *Molds* (*p* = 0.0015), (Table 6).

Histaminogenic flora was not significantly associated with IBS (*p* = 0.7694) or urinary tract infections (*p* = 0.7690), but was significantly linked to alkaline fecal pH (*p* = 0.0001), putrefactive bacteria (*p* < 0.0001), and fungi (*p* = 0.001), suggesting a role in intestinal hyperhistaminemia (Table 7).

Logistic regression identified microbial factors associated with elevated histaminogenic flora, including alkaline fecal pH (OR = 4.01, *p* = 0.0232), reduced acidifying bacteria (*Lactobacillus* spp. and *Bifidobacterium* spp., OR = 1.82, *p* = 0.0232), and fungal overgrowth, particularly *Candida* spp. (OR = 3.98, *p* = 0.0024), suggesting that both bacterial and fungal imbalances contribute to histamine-producing microbiota (Table 8).

Except for frequent fast-food consumption (≥2 times/week, *p* = 0.0267), no significant associations were observed between dietary intake—including whole grains, vegetables, fruits, and water—and histaminogenic flora (Table 9).

## 4. Discussion

These findings are based on culture-dependent data, reflecting only the cultivable component of the microbiota (CCM). The results of this study support the hypothesis that both irritable bowel syndrome (IBS) and urinary tract infections (UTIs) are associated with complex patterns of intestinal dysbiosis [15,16,17]. SLUTIs were linked to an increased prevalence of enteric pathogens alongside a reduction in beneficial microbial taxa, suggesting that intestinal microbial imbalance may contribute to susceptibility to urinary infections. Logistic regression comparing patients with uncomplicated lower urinary tract infections (SLUTIs) to those without infection (NLUTIs) further indicated a broader dysbiotic signature, based on the cultivable component of the microbiota (CCM). Collectively, these results reinforce the concept that microbial dysbiosis plays a meaningful role in the pathogenesis of sporadic lower urinary tract infections. The significant association observed between RLUTIs and altered levels of *Bifidobacterium* spp. supports the hypothesis that disruption of protective commensal populations may predispose individuals to recurrent uropathogen colonization. *Bifidobacterium* spp. plays a key role in maintaining mucosal homeostasis, regulating immune responses, and producing short-chain fatty acids (SCFAs), which contribute to epithelial barrier integrity [18]. Consequently, a reduced abundance of these species may weaken host defense pathways and facilitate uropathogenic persistence.

In our study, individuals without lower urinary tract infections exhibited a more balanced intestinal microbiota, whereas those with sporadic infections showed more pronounced dysbiosis. However, the differences between individuals with sporadic and recurrent infections were less marked. One plausible interpretation is that participants who currently experience only sporadic episodes may already have a microbial imbalance that could, over time, increase their susceptibility to more frequent infections. In this context, persistent dysbiosis may represent an intermediate stage between occasional LUTIs and the progression toward recurrent infection patterns.

In irritable bowel syndrome, opportunistic and histamine-producing bacteria, including *Escherichia coli*, *Enterobacter* spp. and *Clostridium* spp., along with fungal overgrowth (*Candida albicans*, *Candida* spp.), were shown to be increased within the CCM, while protective species such as *Bifidobacterium* spp. and *Lactobacillus* spp. were reduced, indicating an imbalance between commensal and pathogenic microbiota, which may promote mucosal inflammation and gastrointestinal symptoms, consistent with recent evidence on histamine-producing bacteria in visceral hyperalgesia and intestinal inflammation [19,20].

The strong associations identified between histaminogenic flora and specific gut microbiota profiles suggest a relevant microbial signature linked to increased histamine-producing capacity. The significant differences observed for *Escherichia coli*, *Klebsiella* spp., and *Enterobacter* spp. indicate that overgrowth of these taxa may contribute to a pro-inflammatory intestinal environment through enhanced histamine generation or through metabolic pathways that promote mucosal irritation. Many of these genera are known to include opportunistic pathobionts capable of producing biogenic amines or altering epithelial permeability, which may exacerbate histamine-related symptoms and influence host immune responses. These findings align with emerging evidence linking dysbiosis, altered nitrogen metabolism, and heightened histamine activity in conditions such as IBS, food intolerances, and low-grade gut inflammation. Importantly, the results highlight the potential clinical relevance of targeting histaminogenic microbial populations through dietary strategies, prebiotics, or microbiota-modulating interventions aimed at restoring metabolic balance, confirming prior studies on their role in increasing intestinal permeability and systemic inflammation [21,22].

*Candida albicans*, *Candida* spp., *Geotrichum* spp. and *Molds* were consistently associated with histaminogenic flora, emphasizing bacteria–fungi interactions in maintaining a pro-inflammatory gut environment. These findings indicate that elevated histaminogenic flora is strongly associated not only with histamine-producing bacteria but also with overgrowth of opportunistic fungal species. This fungal overrepresentation may exacerbate mucosal inflammation and contribute to dysregulated histamine metabolism in the gut, potentially amplifying intestinal barrier dysfunction and inflammatory responses [23,24,25,26].

Fecal pH may also influence microbiota composition and metabolic responses, with histaminogenic flora associated with an alkaline pH, increased putrefactive bacteria, and fungal presence, suggesting that bacterial metabolism and local intestinal conditions may favor histamine accumulation [27].

Mechanistic Implications. The interplay between histamine-producing bacteria and *Candida albicans* likely contributes to chronic intestinal inflammation, IBS symptomatology, and UTI recurrence. These findings align with recent evidence highlighting the role of complex bacteria–fungi interactions and histamine-producing bacteria in gastrointestinal and extraintestinal disease pathogenesis [28].

The results underscore the central role of intestinal acid-base balance and microbial composition in regulating histaminogenic flora. An alkaline fecal pH, resulting from enhanced proteolytic metabolism of putrefactive bacteria, generates ammonia, biogenic amines, and alkaline metabolites that favor the growth of histamine-producing bacteria. A reduction in acidifying bacteria (*Lactobacillus* spp. and *Bifidobacterium* spp.) decreases the production of organic acids (e.g., lactic, acetic, propionic), which normally maintain a slightly acidic intestinal environment and inhibit pathogenic overgrowth. This loss of protective function promotes dysbiosis and the expansion of decarboxylase-active bacteria involved in histamine metabolism. Fungal overgrowth, particularly *Candida albicans*, further contributes to imbalance by producing alkaline metabolites, consuming fermentable sugars, competing with beneficial bacteria, and indirectly activating inflammatory immune pathways that increase intestinal permeability and histamine absorption. Clinically, these findings suggest that elevated histaminogenic flora reflects complex intestinal dysbiosis, where the interplay between proteolytic bacteria, fungal overgrowth, and depletion of acidifying taxa disrupts intestinal homeostasis. Overall, the logistic regression model indicates that dysbiosis characterized by intestinal alkalinization, reduced acidifying bacteria, and increased fungal load is closely associated with the proliferation of histaminogenic microbiota.

Emerging evidence suggests that alterations in histaminogenic gut flora may not only influence intestinal homeostasis but could also be linked to metabolic disorders, highlighting the potential systemic impact of dysbiosis [29,30,31].

Among all dietary factors assessed, only frequent fast-food consumption, particularly among younger population groups [32], emerged as being associated with elevated histaminogenic flora, whereas inadequate intake of whole grains, vegetables, fruits, or water showed no meaningful relationship with histamine-producing microbial profiles. Direct evidence linking water intake to histaminogenic flora is lacking, but previous studies suggest that both water source and hydration status can influence gut microbial composition and homeostasis, which may indirectly affect the abundance of histamine-producing bacteria [33,34,35].

Recent literature indicates that dietary factors may indirectly affect microbiota and bioactive metabolite production, including histamine, through modulation of fermentation substrates [36]. Accumulating evidence indicates that high-fat diets [35], processed foods, and dietary patterns characteristic of fast-food consumption can disrupt gut microbial homeostasis in ways that may promote the expansion of opportunistic or metabolically active taxa, including bacteria capable of producing biogenic amines such as histamine [36,37,38].

### 4.1. Future Directions

Further research should incorporate advanced sequencing and metabolomics to clarify causal relationships between diet, microbiota, and recurrence risk. Establishing safe thresholds for dietary bioactive amines, along with improving databases on food composition and water intake, is essential for the prevention and integrated management of IBS and recurrent UTIs. Additionally, future studies should employ more detailed dietary assessment tools to examine the broader effects of diet on microbiota composition and related health outcomes and should also carefully control for medication use, given its potential impact on microbiota structure.

Overall, IBS and recurrent LUTIs share a dysbiotic environment dominated by histamine-producing bacteria and *Candida albicans*, promoting chronic inflammation, immune imbalance, and susceptibility to recurrence, emphasizing the clinical relevance of microbiota-targeted interventions.

### 4.2. Limitations

This study did not measure histamine or DAO levels, and microbiota assessment relied solely on culture-based methods, which capture only the cultivable fraction of the flora. Dietary data were obtained through a rapid-screening questionnaire (REAP-S), limiting detailed nutritional analysis. Medication use was based on self-report, although participants using antibiotics or other microbiota-modifying drugs were excluded.

## 5. Conclusions

These conclusions are derived from culture-dependent data and therefore reflect only the cultivable component of the microbiota (CCM). In this observational study, lower urinary tract infections (LUTIs) and irritable bowel syndrome (IBS) were associated with an increased presence of histaminogenic flora, characterized by histamine-producing bacteria (e.g., *Escherichia coli*, *Klebsiella* spp., *Enterobacter* spp.) and fungi (*Candida albicans*), alongside a reduced presence of microorganisms commonly regarded as beneficial, such as *Lactobacillus* and *Bifidobacterium*. This microbial pattern was associated with an alkaline fecal pH and increased prevalence of putrefactive bacteria. While general dietary patterns and fluid intake showed limited associations, frequent fast-food consumption was associated with higher levels of histaminogenic flora. Associations observed in this study suggest that strategies supporting beneficial microbiota—such as prebiotics, probiotics, and dietary components favoring *Lactobacillus* and *Bifidobacterium*—may be relevant for the management of IBS and recurrent LUTIs, although causal relationships cannot be inferred from these data.

## Figures and Tables

**Table 1:** Relationship Between Gut Microbiota and Clinical LUTI Subgroups: absence of LUTIs, sporadic, and recurrent.

Bacterial Populations	Microorganisms	LUTIs		*p*-Value	LUTIs		*p*-Value
		NLUTIs (*n* = 19)	SLUTIs (*n* = 117)		SLUTIs (*n* = 117)	RLUTIs (*n* = 52)	
Putrefactive flora	*Escherichia coli*			0.0143			0.5026
	normal	4 (21.05%)	6 (5.12%)		6 (5.12%)	1 (1.92%)	
	high	13 (68.42%)	107 (91.45%)		107 (91.45%)	48 (92.30%)	
	low	2 (10.52%)	4 (3.41%)		4 (3.41%)	3 (5.76%)	
	*Proteus* spp.			0.5836			0.6677
	normal	1 (5.26%)	112 (95.72%)		112 (95.72%)	51 (98.07%)	
	high	17 (89.47%)	5 (4.27%)		5 (4.27%)	1 (1.92%)	
	*Klebsiella* spp.			0.297			0.6727
	normal	17 (89.47%)	99 (84.61%)		99 (84.61%)	46 (76.92%)	
	high	2 (10.52%)	18 (15.38%)		18 (15.38%)	6 (11.53%)	
	*Enterobacter* spp.			0.197			0.9952
	normal	17 (89.47%)	103 (88.03%)		103 (88.03%)	43 (82.69%)	
	high	2 (10.52%)	13 (11.11%)		13 (11.11%)	8 (15.38%)	
	low	0 (0%)	1 (0.85%)		1 (0.85%)	1 (1.92%)	
	*Clostridium* spp.			0.2862			0.4286
	normal	18 (94.73%)	106 (90.59%)		106 (90.59%)	47 (90.38%)	
	high	0 (0%)	8 (6.83%)		8 (6.83%)	5 (9.61%)	
	low	1 (5.26%)	3 (2.56%)		3 (2.56%)	0 (0%)	
Acidifying flora	*Bifidobacterium* spp.			<0.0001			<0.0001
	normal	14 (73.68%)	20 (17.09%)		20 (17.09%)	1 (1.92%)	
	high	5 (26.31%)	0 (0%)		0 (0%)	11 (21.15%)	
	low	0 (0%)	97 (82.90%)		97 (82.90%)	40 (76.92%)	
	*Lactobacillus* spp.			0.0203			0.1508
	normal	6 (31.57%)	58 (49.57%)		58 (49.57%)	20 (38.46%)	
	high	1 (5.26%)	0 (0%)		0 (0%)	1 (1.92%)	
	low	12 (63.15%)	59 (50.42%)		59 (50.42%)	31 (59.61%)	
Yeasts	*Candida albicans*			<0.0001			0.6206
	normal	17 (89.47%)	33 (28.20%)		33 (28.20%)	13 (25%)	
	high	2 (10.52%)	82 (70.08%)		82 (70.08%)	39 (75%)	
	*Candida* spp.			<0.0001			0.9677
	normal	17 (89.47%)	22 (18.80%)		22 (18.80%)	10 (19.20%)	
	high	2 (10.52%)	94 (80.34%)		94 (80.34%)	42 (80.76)	
	*Geotrichum* spp.			0.594			0.9845
	normal	15 (78.94%)	103 (88.03%)		103 (88.03%)	45 (86.53%)	
	high	4 (21.05%)	14 (11.96%)		14 (11.96%)	7 (13.46%)	

**Table 2:** Association Between Microbiota Alterations and the Risk of Sporadic Lower Urinary Tract Infections (SLUTIs): Logistic Regression Analysis.

Variables	NLUTIs vs. SLUTIs		
	OR	95% CI	*p*-Value
Increased *Escherichia coli*	1.7822	1.2640 to 2.3179	0.0357
Increased *Klebsiella* spp.	1.4481	0.2875 to 7.2922	0.8686
Increased *Clostridium* spp.	0.9287	0.1205 to 7.1589	0.9133
Decreased *Lactobacillus*_spp.	0.7208	0.4149 to 1.2523	0.1727
Decreased *Bifidobacterium* spp.	0.7360	0.4054 to 1.3361	0.3136
Increased *Candida_albicans*	3.6587	2.7872 to 17.0044	0.0123

**Table 3:** Association Between Microbiota Alterations and the Risk of Recurrent Lower Urinary Tract Infections (RLUTIs): Logistic Regression Analysis.

Variables	NLUTIs vs. SLUTIs		
	OR	95% CI	*p*-Value
Increased *Escherichia coli*	2.1616	1.9647 to 4.8433	0.0611
Increased *Klebsiella* spp.	0.7015	0.2506 to 1.9635	0.4996
Increased *Clostridium*_spp.	1.1065	0.3716 to 3.2946	0.8557
Decreased *Lactobacillus*_spp.	1.7796	1.5520 to 3.1011	0.0228
Decreased *Bifidobacterium* spp.	1.0828	0.7100 to 1.6514	0.7117
Increased *Candida_albicans*	0.6347	0.3042 to 1.3243	0.2257

**Table 4:** Associations Between Sporadic LUTIs in Women with IBS and Gut Microbiota Composition.

Bacterial Populations	Microorganisms	SLUTI-IBS		*p*-Value
		SLUTI-NIBS (*n* = 70)	SLUTI-IBS (*n* = 47)	
Putrefactive flora	*Escherichia coli*			<0.0001
	normal	51 (72.85%)	10 (21.27%)	
	high	18 (25.71%)	36 (76.59%)	
	low	1 (1.42%)	1 (2.12%)	
	*Proteus* spp.			0.645
	normal	70 (100%)	43 (91.48%)	
	high	0 (0%)	4 (8.51%)	
	*Klebsiella* spp.			0.297
	normal	60 (85.71%)	40 (85.10%)	
	high	10 (1.42%)	7 (14.89%)	
	*Enterobacter* spp.			0.008
	normal	63 (90%)	41 (87.23%)	
	high	7 (10%)	5 (10.63%)	
	low	0 (0%)	1 (2.12%)	
	*Clostridium* spp.			0.006
	normal	65 (92.85%)	44 (93.61%)	
	high	4 (5.71%)	3 (6.38%)	
	low	1 (1.42%)	0 (0%)	
Acidifying flora	*Bifidobacterium* spp.			<0.0001
	normal	60 (85.71%)	23 (48.93%)	
	high	1 (1.42%)	0 (0%)	
	low	9 (12.85%)	24 (51.06%)	
	*Lactobacillus* spp.			0.0224
	normal	36 (511.42%)	17 (36.17%)	
	high	5 (7.14%)	0 (0%)	
	low	29 (41.42%)	30 (63.82%)	
Yeasts	*Candida albicans*			<0.0001
	normal	49 (70%)	15 (31.91%)	
	high	21 (30%)	32 (68.08%)	
	*Candida* spp.			<0.0001
	normal	54 (77.14%)	6 (12.76%)	
	high	16 (22.85%)	41 (87.23%)	
	*Geotrichum* spp.			0.154
	normal	66 (94.28%)	26 (55.31%)	
	high	4 (57.14%)	19 (40.42%)	

**Table 5:** Bacterial species distribution across different levels of histaminogenic flora.

Microorganisms	Histaminogenic Flora		*p*-Value
	NHF (*n* = 36)	IHF (*n* = 152)	
*Escherichia coli*			<0.0001
normal	28 (77.77%)	9 (59.21%)	
high	2 (5.55%)	142 (93.42%)	
low	4 (11.11%)	3 (1.97%)	
*Klebsiella* spp.			<0.0001
normal	35 (97.22%)	107 (70.39%)	
high	0 (0%)	46 (30.26%)	
*Enterobacter* spp.			<0.0001
normal	34 (94.44%)	106 (69.73%)	
high	0 (0%)	48 (31.57%)	
*Proteus* spp.			0.9999
normal	33 (91.66%)	147 (96.71%)	
high	1 (2.77%)	5 (3.28%)	
*Clostridium* spp.			0.2759
normal	34 (94.44%)	144 (94.73)	
high	1 (2.77%)	7 (4.60%)	
low	1 (2.77%)	1 (0.65%)	

**Table 6:** Fungal species distribution across different levels of histaminogenic flora.

Microorganisms	Histaminogenic Flora		*p*-Value
	NHF (*n* = 36)	IHF(*n* = 152)	
*Candida albicans*			<0.0001
normal	30 (83.33%)	48 (31.57%)	
high	1 (2.77%)	105 (69.07%)	
low	1 (2.77%)	1 (0.65%)	
*Candida* spp.			<0.0001
normal	21 (58.33%)	36 (23.36%)	
high	6 (16.66%)	125 (82.23%)	
*Geotrichum* spp.			<0.0001
normal	35 (97.22%)	112 (73.68%)	
high	1 (2.77%)	40 (26.31%)	
*Molds*			0.0015
normal	34 (94.44%)	116 (76.31%)	
high	1 (2.77%)	37 (24.32%)	

**Table 7:** Distribution of histaminogenic flora across clinical and microbial parameters.

Microorganisms	Histaminogenic Flora		*p*-Value
	NHF (*n* = 36)	IHF (*n* = 152)	
IBS			0.7694
no	26 (72.22%)	106 (69.73%)	
yes	10 (27.77%)	46 (30.26%)	
LUTIs (SLUTIs and RLUTIs)			0.7690
no	4 (11.11%)	15 (9.86%)	
yes	32 (88.88%)	133 (87.5%)	
Fecal pH fecal			0.0001
Normal	26 (72.22%)	61 (40.13%)	
Alkaline	8 (22.22%)	93 (61.18%)	
Increased putrefactive flora			<0.0001
normal	33 (91.66%)	0 (0%)	
high	1 (2.77%)	152 (100%)	
Decreased acidifying flora			0.050
normal	9 (25)	18 (11.84)	
low	15 (41.66)	144 (94.73)	
Yeasts			0.001
normal	25 (69.44%)	62 (40.78%)	
high	9 (25%)	90 (59.21%)	

**Table 8:** Microbial Factors Associated with Increased Histaminogenic Flora: Logistic Regression Results.

Variables	Increased Histaminogenic Flora		
	OR	95% CI	*p*-Value
Alkaline fecal pH (increased putrefactive flora)	4.0062	1.6499 to 9.7280	0.0232
Decreased acidifying flora	1.8159	1.0851 to 3.0391	0.0232
Increased yeast flora	3.9817	1.6310 to 9.7204	0.0024

**Table 9:** Associations between dietary intake and histaminogenic flora levels.

Dietary Intake	Histaminogenic Flora		*p*-Value
	NHF (*n* = 36)	IHF (*n* = 152)	
Fewer than 3 servings of whole grains per day			0.084
1	17 (47.22%)	50 (32.89%)	
2	7 (19.44%)	32 (21.05%)	
3	3 (8.33%)	39 (25.65%)	
4	1 (2.77%)	12 (7.89%)	
5	5 (13.88%)	20 (13.15%)	
Fewer than 3 servings of vegetables (excluding potatoes) per day			0.614
1	9 (25%)	45 (29.60%)	
2	6 (16.66%)	25 (16.44%)	
3	12 (33.33%)	36 (23.68%)	
4	3 (8.33%)	18 (11.84%)	
5	5 (13.88%)	27 (17.76%)	
Fewer than 2 servings of fruit per day			0.684
1	9 (25%)	49 (32.23%)	
2	10 (27.77%)	28 (52.63%)	
3	6 (16.66%)	35 (23.02%)	
4	6 (16.66%)	11 (7.23%)	
5	6 (16.66%)	28 (18.42%)	
Fast food consumption twice a week or more			0.0267
1	23 (63.88%)	99 (65.13)	
2	5 (13.88%)	6 (3.94)	
3	4 (11.11%)	11 (7.23)	
4	1 (2.77%)	21 (13.81)	
5	1 (2.77%)	17 (11.18)	
Less than 1 L of water per day			0.924
1	18 (50%)	71 (46.71%)	
2	2 (5.55%)	12 (7.89%)	
3	6 (16.66%)	27 (17.76%)	
4	2 (5.55%)	14 (9.21%)	
5	6 (16.66%)	22 (14.47%)	

## Data Availability

The data that support the findings of this study are available from the corresponding author upon reasonable request.
